# Supplementary material for: Do political incentives promote or inhibit corporate social responsibility? The role of local officials’ tenure
Source: PLoS One. 2023 Mar 17;18(3):e0283183. doi: 10.1371/journal.pone.0283183 (PMC10022816; doi:10.1371/journal.pone.0283183)
Supplement: S2 Table — (DOC) [file pone.0283183.s002.doc]

# S2 Table

**Correlation metrics.**

| **Panel A: Heckman first-stage variables** | | | | | | | | | | | | | |
| --- | --- | --- | --- | --- | --- | --- | --- | --- | --- | --- | --- | --- | --- |
| **Variables** | **(1)** | **(2)** | **(3)** | **(4)** | **(5)** | **(6)** | **(7)** | **(8)** | **(9)** | **(10)** | **(11)** | **(12)** | **(13)** |
| (1) Report |  |  |  |  |  |  |  |  |  |  |  |  |  |
| (2) Policy | 0.690*** |  |  |  |  |  |  |  |  |  |  |  |  |
| (3) Slack resource | 0.086*** | 0.077*** |  |  |  |  |  |  |  |  |  |  |  |
| (4) ROA | 0.087*** | 0.091*** | 0.263*** |  |  |  |  |  |  |  |  |  |  |
| (5) Leverage | 0.143*** | 0.159*** | -0.147*** | -0.175*** |  |  |  |  |  |  |  |  |  |
| (6) Firm age | 0.186*** | 0.181*** | -0.026*** | -0.127*** | 0.389*** |  |  |  |  |  |  |  |  |
| (7) Firm size | 0.448*** | 0.443*** | 0.061*** | 0.102*** | 0.435*** | 0.327*** |  |  |  |  |  |  |  |
| (8) Foreign income | -0.026*** | -0.036*** | 0.025*** | -0.008 | -0.064*** | -0.131*** | -0.025*** |  |  |  |  |  |  |
| (9) Equity concentration | 0.039*** | -0.002 | 0.104*** | 0.233*** | -0.174*** | -0.403*** | 0.134*** | 0.052*** |  |  |  |  |  |
| (10) Board size | 0.131*** | 0.128*** | 0.028*** | -0.046*** | 0.154*** | 0.168*** | 0.248*** | -0.036*** | -0.014** |  |  |  |  |
| (11) Board independence | -0.005 | 0.001 | -0.006 | 0.024*** | -0.062*** | -0.101*** | -0.023*** | 0.037*** | 0.046*** | -0.127*** |  |  |  |
| (12) Female executive | -0.046*** | -0.040*** | -0.024*** | 0.009 | -0.034*** | -0.032*** | -0.074*** | 0.009 | -0.022*** | 0.023*** | 0.030*** |  |  |
| (13) Executive age | 0.188*** | 0.196*** | 0.072*** | 0 | 0.109*** | 0.238*** | 0.333*** | -0.041*** | -0.008 | 0.195*** | 0.008 | -0.054*** |  |
| (14) Industry-level CSR | 0.080*** | 0.053*** | 0.001 | -0.077*** | 0.126*** | 0.295*** | 0.225*** | 0.013** | -0.137*** | 0.099*** | 0.013** | -0.054*** | 0.143*** |
| **Panel B: Heckman second-stage variables** | | | | | | | | | | | | | |
| **Variables** | **(1)** | **(2)** | **(3)** | **(4)** | **(5)** | **(6)** | **(7)** | **(8)** | **(9)** | **(10)** | **(11)** | **(12)** | **(13)** |
| (1) CSR score |  |  |  |  |  |  |  |  |  |  |  |  |  |
| (2) Tenure of governor | -0.054*** |  |  |  |  |  |  |  |  |  |  |  |  |
| (3) Tenure of party secretary | -0.014 | 0.244*** |  |  |  |  |  |  |  |  |  |  |  |
| (4) GDP priority | -0.287*** | 0.147*** | 0.022* |  |  |  |  |  |  |  |  |  |  |
| (5) MarketDev | 0.166*** | 0.053*** | 0.017 | -0.508*** |  |  |  |  |  |  |  |  |  |
| (6) Slack resource | 0.076*** | -0.005 | 0.014 | 0.009 | -0.013 |  |  |  |  |  |  |  |  |
| (7) ROA | 0.041*** | 0.065*** | 0.040*** | 0.01 | 0.074*** | 0.325*** |  |  |  |  |  |  |  |
| (8) Leverage | 0.124*** | 0.02 | 0.019 | 0.002 | -0.014 | -0.229*** | -0.152*** |  |  |  |  |  |  |
| (9) Firm age | 0.091*** | -0.054*** | -0.072*** | -0.183*** | 0.029** | -0.060*** | -0.118*** | 0.224*** |  |  |  |  |  |
| (10) Firm size | 0.468*** | -0.036*** | 0.027** | -0.195*** | 0.089*** | -0.005 | 0.071*** | 0.520*** | 0.245*** |  |  |  |  |
| (11) Foreign income | 0.033*** | 0.014 | -0.006 | -0.071*** | 0.099*** | 0.016 | -0.021* | -0.008 | -0.095*** | -0.026** |  |  |  |
| (12) SOE | 0.135*** | -0.001 | 0.058*** | 0.075*** | -0.136*** | -0.018 | -0.109*** | 0.233*** | 0.288*** | 0.307*** | -0.126*** |  |  |
| (13) Equity concentration | 0.252*** | -0.026** | 0.035*** | -0.074*** | 0.086*** | 0.125*** | 0.147*** | -0.001 | -0.300*** | 0.277*** | -0.013 | 0.098*** |  |
| (14) Board size | 0.162*** | -0.038*** | -0.001 | -0.029** | -0.060*** | 0.029** | -0.060*** | 0.117*** | 0.096*** | 0.234*** | -0.023* | 0.235*** | 0.065*** |
| (15) Board independence | 0.025** | -0.008 | 0.019 | 0.003 | 0.043*** | 0 | 0.031** | 0.001 | -0.075*** | 0.040*** | 0.032** | -0.103*** | 0.029** |
| (16) Female executive | -0.018 | 0.045*** | 0.100*** | 0.01 | 0.068*** | -0.028** | 0.01 | -0.034*** | -0.027** | -0.083*** | -0.005 | -0.071*** | -0.074*** |
| (17) Executive age | 0.281*** | -0.032** | 0.034*** | -0.118*** | 0.099*** | 0.004 | -0.046*** | 0.148*** | 0.199*** | 0.407*** | -0.036*** | 0.363*** | 0.161*** |
| (18) GDP per capita | 0.240*** | -0.022* | 0.038*** | -0.599*** | 0.816*** | -0.044*** | -0.002 | 0 | 0.135*** | 0.185*** | 0.077*** | -0.075*** | 0.106*** |
| (19) Population growth | 0.008 | -0.016 | 0.072*** | -0.009 | -0.250*** | 0.012 | -0.023* | -0.034*** | -0.027** | -0.071*** | 0.070*** | -0.102*** | -0.066*** |
| (20) Fiscal revenue | 0.279*** | 0.004 | -0.02 | -0.567*** | 0.825*** | 0.01 | 0.068*** | -0.003 | 0.092*** | 0.182*** | 0.106*** | -0.131*** | 0.078*** |
| (21) Industry-level CSR | 0.224*** | -0.049*** | -0.025* | -0.251*** | 0.137*** | -0.025** | -0.053*** | 0.055*** | 0.211*** | 0.174*** | 0.033*** | 0.044*** | -0.018 |
| (22) Age of governor | 0.004 | 0.385*** | 0.323*** | 0.012 | 0.040*** | 0.013 | 0.039*** | 0.008 | -0.022* | -0.005 | -0.025* | 0.060*** | 0.009 |
| (23) Age of party secretary | -0.02 | 0.004 | 0.336*** | -0.135*** | 0.110*** | -0.018 | -0.047*** | 0.025** | -0.021* | 0.071*** | -0.036*** | 0.080*** | 0.097*** |
| (24) Education of governor | 0.160*** | -0.167*** | -0.239*** | -0.267*** | 0.042*** | -0.014 | -0.059*** | 0.002 | 0.154*** | 0.106*** | 0.044*** | -0.067*** | -0.025* |
| (25) Education of party secretary | 0.025** | -0.176*** | -0.112*** | 0.027** | -0.173*** | 0.033*** | -0.041*** | -0.023* | 0.084*** | 0.030** | -0.008 | 0.001 | -0.005 |
| (26) Birthplace of governor | -0.124*** | 0.288*** | -0.098*** | 0.087*** | -0.167*** | 0.046*** | 0.067*** | -0.017 | -0.093*** | -0.099*** | 0.066*** | -0.102*** | -0.049*** |
| (27) Birthplace of party secretary | -0.049*** | -0.031** | 0.217*** | 0.205*** | -0.122*** | 0.038*** | 0.038*** | -0.029** | -0.027** | 0.014 | 0.007 | -0.038*** | -0.030** |
| **Variables** | **(14)** | **(15)** | **(16)** | **(17)** | **(18)** | **(19)** | **(20)** | **(21)** | **(22)** | **(23)** | **(24)** | **(25)** | **(26)** |
| (15) Board independence | -0.104*** |  |  |  |  |  |  |  |  |  |  |  |  |
| (16) Female executive | 0.017 | 0.036*** |  |  |  |  |  |  |  |  |  |  |  |
| (17) Executive age | 0.195*** | 0.080*** | -0.094*** |  |  |  |  |  |  |  |  |  |  |
| (18) GDP per capita | -0.031** | 0.052*** | 0.025** | 0.178*** |  |  |  |  |  |  |  |  |  |
| (19) Population growth | 0.028** | -0.026** | 0.028** | -0.129*** | -0.319*** |  |  |  |  |  |  |  |  |
| (20) Fiscal revenue | 0 | 0.030** | 0.034*** | 0.102*** | 0.719*** | -0.118*** |  |  |  |  |  |  |  |
| (21) Industry-level CSR | 0.048*** | 0.014 | -0.045*** | 0.143*** | 0.250*** | -0.003 | 0.240*** |  |  |  |  |  |  |
| (22) Age of governor | 0.01 | -0.003 | 0.060*** | 0.009 | -0.032** | -0.177*** | 0.051*** | -0.034*** |  |  |  |  |  |
| (23) Age of party secretary | -0.011 | 0.031** | -0.004 | 0.127*** | 0.310*** | -0.249*** | -0.059*** | 0.063*** | -0.01 |  |  |  |  |
| (24) Education of governor | 0.057*** | -0.034*** | -0.060*** | 0.017 | 0.140*** | 0.193*** | 0.180*** | 0.155*** | -0.398*** | -0.062*** |  |  |  |
| (25) Education of party secretary | 0.028** | -0.003 | -0.059*** | 0.021* | -0.032** | -0.018 | -0.059*** | 0.108*** | -0.084*** | -0.046*** | 0.030** |  |  |
| (26) Birthplace of governor | -0.046*** | -0.051*** | 0.022* | -0.134*** | -0.218*** | 0.121*** | -0.121*** | -0.118*** | 0.104*** | -0.188*** | -0.189*** | 0.281*** |  |
| (27) Birthplace of party secretary | 0.028** | -0.013 | 0.021* | -0.044*** | -0.125*** | 0.081*** | -0.007 | -0.053*** | -0.044*** | -0.094*** | -0.019 | 0.012 | 0.092*** |

Note. *** p<0.01, ** p<0.05, * p<0.1
