# Supplementary material for: Do political incentives promote or inhibit corporate social responsibility? The role of local officials’ tenure
Source: PLoS One. 2023 Mar 17;18(3):e0283183. doi: 10.1371/journal.pone.0283183 (PMC10022816; doi:10.1371/journal.pone.0283183)
Supplement: S3 Table — (DOC) [file pone.0283183.s003.doc]

# S3 Table

**Statistical tests of the U-shaped relationship between the government leader’s tenure and CSR.**

| Panel C | | | | |
| --- | --- | --- | --- | --- |
| Squared term significant | Turning point | lCL | uCL | Within bounds |
| yes | 4.10 | 3.04 | 6.22 | yes |
| Panel D | | | | |
| Lower bound: t (p) | Upper bound: t (p) | Overall t-statistic | Overall p-value |  |
| -3.00(0.00) | 2.45(0.01) | 2.45 | 0.01 |  |

Notes. Panel C reports whether the squared term is significant at the 0.05 level at least. Turning point – turning point in years. lCL and uCL – the lower and upper confidence intervals for the turning point, respectively, at 95% confidence level. Within bounds – whether the turning point is within bounds of the data, where the minimum and maximum values of the governor’s tenure are 1 and 9, respectively. The turning point is within bounds if the lCL and uCL are above or below the minimum and maximum values. Panel D reports the results of the Sasabuchi test for the presence of a U-shaped relationship. Lower bound: t (p) shows the t-statistic and associated p-value.
