# Supplementary material for: Do political incentives promote or inhibit corporate social responsibility? The role of local officials’ tenure
Source: PLoS One. 2023 Mar 17;18(3):e0283183. doi: 10.1371/journal.pone.0283183 (PMC10022816; doi:10.1371/journal.pone.0283183)
Supplement: S4 Table — (DOC) [file pone.0283183.s004.doc]

S4 Table

**Robustness checks with the standard errors clustered by provinces.**

| **Model No.** | **Model 1** | **Model 2** | **Model 3** | **Model 4** |
| --- | --- | --- | --- | --- |
| Tenure of governor | -0.728** | 0.053 | -0.811** | -0.237 |
|  | (-2.294) | (0.092) | (-2.411) | (-0.438) |
| Tenure of governor^2^ (H1) | 0.089** | 0.009 | 0.101** | 0.046 |
|  | (2.404) | (0.165) | (2.497) | (0.820) |
| Tenure of party secretary | 0.061 | 0.290 | 0.022 | 0.241 |
|  | (0.255) | (1.153) | (0.087) | (0.971) |
| Tenure of party secretary^2^ (H2) | 0.019 | -0.011 | 0.022 | -0.009 |
|  | (0.712) | (-0.404) | (0.764) | (-0.311) |
| GDP priority |  | -1.992*** |  | -2.338*** |
|  |  | (-3.870) |  | (-3.760) |
| Tenure of governor*GDP priority |  | -1.296** |  | -0.780 |
|  |  | (-2.495) |  | (-1.393) |
| Tenure of governor^2^*GDP priority (H3) |  | 0.152*** |  | 0.092 |
|  |  | (2.829) |  | (1.528) |
| MarketDev |  |  | -0.749 | -0.864* |
|  |  |  | (-1.621) | (-1.855) |
| Tenure of governor*MarketDev |  |  | 0.239* | 0.261* |
|  |  |  | (1.860) | (1.786) |
| Tenure of governor^2^*MarketDev (H4) |  |  | -0.031 | -0.037* |
|  |  |  | (-1.678) | (-1.800) |
| Slack resource | 9.208*** | 9.139*** | 9.314*** | 9.194*** |
|  | (4.067) | (3.896) | (4.099) | (3.948) |
| ROA | -0.115 | -0.255 | 0.037 | -0.072 |
|  | (-0.054) | (-0.120) | (0.017) | (-0.034) |
| Leverage | -5.879*** | -5.739*** | -5.789*** | -5.619*** |
|  | (-4.345) | (-4.263) | (-4.251) | (-4.143) |
| Firm age | -0.051 | -0.056 | -0.048 | -0.054 |
|  | (-1.019) | (-1.145) | (-0.992) | (-1.135) |
| Firm size | 4.139*** | 4.101*** | 4.107*** | 4.059*** |
|  | (10.084) | (9.861) | (9.766) | (9.513) |
| Foreign income | 0.775 | 0.807 | 0.861 | 0.902 |
|  | (0.623) | (0.656) | (0.684) | (0.724) |
| SOE | 1.378* | 1.389* | 1.270* | 1.264* |
|  | (1.809) | (1.787) | (1.731) | (1.701) |
| Equity concentration | 0.070*** | 0.068*** | 0.070*** | 0.069*** |
|  | (3.673) | (3.587) | (3.711) | (3.613) |
| Board size | 0.121 | 0.113 | 0.117 | 0.109 |
|  | (1.217) | (1.134) | (1.176) | (1.087) |
| Board independence | -1.217 | -1.049 | -1.378 | -1.223 |
|  | (-0.552) | (-0.493) | (-0.603) | (-0.551) |
| Female executive | 1.236*** | 1.255*** | 1.233*** | 1.256*** |
|  | (2.881) | (3.015) | (2.922) | (3.059) |
| Executive age | 0.253** | 0.252** | 0.260*** | 0.260*** |
|  | (2.717) | (2.689) | (2.814) | (2.817) |
| GDP per capita | 0.162 | -0.284 | 1.552 | 1.228 |
|  | (0.092) | (-0.177) | (0.955) | (0.811) |
| Population growth | 0.129 | 0.093 | 0.134 | 0.098 |
|  | (0.671) | (0.499) | (0.803) | (0.613) |
| Fiscal revenue | 1.168 | 0.850 | 2.037* | 1.829* |
|  | (1.528) | (1.142) | (1.919) | (1.848) |
| Industry-level CSR | 0.030*** | 0.031*** | 0.032*** | 0.034*** |
|  | (3.293) | (3.565) | (3.570) | (3.884) |
| Age of governor | 0.094 | 0.064 | 0.082 | 0.052 |
|  | (1.220) | (0.886) | (1.040) | (0.707) |
| Age of party secretary | -0.258*** | -0.266*** | -0.259*** | -0.266*** |
|  | (-3.221) | (-3.275) | (-3.330) | (-3.290) |
| Education of governor | 0.769 | 0.623 | 0.694 | 0.555 |
|  | (1.162) | (1.048) | (1.234) | (1.127) |
| Education of party secretary | -0.291 | -0.082 | -0.414 | -0.159 |
|  | (-0.719) | (-0.222) | (-1.021) | (-0.438) |
| Birthplace of governor | -0.365 | -0.836 | -0.450 | -1.033 |
|  | (-0.530) | (-1.095) | (-0.649) | (-1.413) |
| Birthplace of party secretary | -2.350** | -1.577* | -2.736** | -1.940** |
|  | (-2.474) | (-1.854) | (-2.572) | (-2.130) |
| IMR | 0.628*** | 0.642*** | 0.656*** | 0.673*** |
|  | (2.810) | (2.905) | (3.080) | (3.186) |
| Constant | -87.525*** | -74.229*** | -116.984*** | -105.861*** |
|  | (-4.888) | (-3.904) | (-5.179) | (-4.944) |
| Industry FE | yes | yes | yes | yes |
| Year FE | yes | yes | yes | yes |
| Observations | 6319 | 6319 | 6319 | 6319 |
| R-squared | 0.363 | 0.366 | 0.364 | 0.368 |
| F | 209.995 | 13688.591 | 553.020 |  |

Note. Correlation metrics of all variables in the second stage are represented in S2 Table. Robust t-statistics (in parentheses) are based on the standard errors clustered by provinces to address potential serial correlations in the residuals. F statistic in Model 4 is missing due to collinearity or too few clusters. *** p<0.01, ** p<0.05, * p<0.1.
